# Indoor occupational risk assessment of traffic-related nitrogen dioxide in public hospitals, Alexandria, Egypt

**DOI:** 10.1186/s42506-025-00202-1

**Published:** 2026-02-13

**Authors:** Eman M. A. Abd El-Latef, Abd-AlAziz Kamel, Gehan R. Zaki, Ahmed I. Issa, Amira Abdelraheem

**Affiliations:** 1 Occupational Hygiene and Air Pollution, Environmental Health Department, High Institute of Public Health, Alexandria University, Alexandria, Egypt; 2 Occupational Health and Industrial Medicine, Occupational Health and Industrial Medicine Department, High Institute of Public Health, Alexandria University, Alexandria, Egypt

**Keywords:** Air quality, Nitrogen dioxide, Occupational risk assessment, Hospitals

## Abstract

**Background:**

The U.S. Environmental Protection Agency ranks indoor air quality as one of the most prominent environmental problems However, limited research has addressed indoor air quality in Egypt, particularly in highly sensitive environments such as hospitals. Moreover, traffic-related pollutants such as nitrogen dioxide (NO₂) are rarely investigated as risk factors in hospitals. Therefore, this study assessed the occupational exposure risks associated with traffic-related NO₂ in four public hospitals on different main streets in Alexandria, Egypt.

**Methods:**

This study was carried out through indoor nitrogen dioxide sampling during the morning shifts in four public hospitals in Alexandria, Egypt. Hospitals were selected and categorized based on the traffic congestion levels of their main streets. The traffic congestion categories included heavy, moderate, and low congestion areas as well as on-sea road areas. One hospital was randomly chosen from each category.

**Results:**

The results of the present study showed the highest NO₂ exposure recorded among hospital staff located in the heavy traffic area during hot months, whereas the lowest exposure observed was among on-sea hospital staff. The results indicated that outdoor traffic emissions were the primary source of indoor NO₂ exposure. The results showed a strong and statistically significant correlation with nearby traffic congestion. The risk of indoor nitrogen dioxide concentrations during hot and cold months was high at most indoor locations: 72.7% and 63.6% of locations in the heavy traffic hospital during hot and cold months, respectively. Moderate risk was observed at most of the locations at moderate traffic hospital (77.8%), the sea-road hospital (57.1%), and the low traffic hospital (54.5%) during both hot and cold months.

**Conclusions:**

This study concludes that NO₂ concentrations increase with traffic congestion, and the risk factor was found to be moderate to high at most of the measured indoor locations in the four hospitals. It is recommended to reduce NO₂ exposure among hospital staff and patients by utilizing mechanical ventilation systems with air filtration.

## Introduction

Hospitals are a major part of the total healthcare system. They are supposed to function efficiently and effectively to achieve Sustainable Development Goal (SDG 3):“Ensure healthy lives and promote well-being for all at all ages” [1, 2].

The hospital environment is unique and complex, differing from other commercial or residential buildings. An Iranian review in 2017 considered environmental conditions, including ambient air quality and wind direction, to be the major factors affecting proper hospital site selection [3]. Therefore, traffic activity, as a major outdoor source, may affect hospital indoor air quality (H-IAQ). H-IAQ is especially important for the inpatients and outpatients, the staff caring for them, and visitors. Poor H-IAQ may exacerbate the patients’ vulnerability to further health complications [1]. In addition, it may contribute sick building syndrome (SBS) in healthy individuals, leading to headaches, fatigue, trouble concentrating, and irritation of the eyes, nose, throat, and lungs. Ensuring good H-IAQ is a key objective for improving occupants’ health and satisfaction [4–6]. Therefore, the H-IAQ concept is emerging as a modern field of specialization among engineers and health care professionals [1].

There are many chemical species of nitrogen oxides, but from a health and chemical stability perspective, nitrogen dioxide (NO₂)is the most important. The main sources of NO₂ in the indoor environment are traffic and kitchen activities [7]. The World Health Organization and many researchers have found that indoor exposure to NO₂ may lead to various adverse health effects, including irritation of the eyes, nose, throat, and respiratory tract[8]. Many studies have investigated the health effects of NO₂ using both experimental animals and controlled human clinical studies on both healthy and sensitive populations[8].

 The results of some of these studies found that NO₂ concentrations below 500 µg/m³ have no significant health effects on healthy individuals; however, cough, sneezing, and other symptoms may be exacerbated in asthmatics [8]. Concentrations ranging from 500 to 2000 µg/m³ may lead to an increased prevalence of acute respiratory symptoms and a reduction in pulmonary function, particularly during physical activity, as evidenced by decreased forced expiratory volume (FEV₁). Additionally, there is an increased prevalence of respiratory complaints, chest tightness, cough, and wheezing, particularly in asthmatics [8, 9]. Conversely, for NO₂ concentrations above 2000 µg/m³, studies have reported an increased prevalence of acute bronchitis and bronchopneumonia, reduced arterial oxygen pressure, and increased airway resistance, as manifested by a decrease in forced vital capacity (FVC), FEV1, and FEV1/FVC ratio (as detected by pulmonary function tests (PFT)) [8–10].

Risk assessment (RA) is a tool used to study the likelihood and severity of undesirable event(s). It describes the overall process of identifying, evaluating, and characterizing the risk. RA provides a comprehensive evaluation of the workplace to identify exposures, situations, and processes that may cause harm. Then, it involves analyzing and evaluating the likelihood and severity of these risks to calculate risk factors. Based on the calculated risk factors, appropriate control measures are determined to minimize the risk [11, 12].

To the best of our knowledge, there is a lack of research addressing indoor air quality in Egypt, especially in highly sensitive environments such as hospitals. In addition, traffic air pollutants such as NO₂ are rarely studied as risk factors in hospitals. This study aims to assess the occupational risks of NO₂ traffic pollution on hospital indoor air quality across various locations within public hospitals during different seasons.

## Methods

### Study design

This cross-sectional study was conducted during the hot and cold seasons from August 2020 to January 2021. The study was performed at four public hospitals in Alexandria, which were selected based on their traffic characteristics.

### Sampling design

The selection of hospitals was based on classifying Alexandria into regions according to the traffic congestion levels of the main streets. This classification was determined by categorizing vehicle speeds, with each class representing a traffic congestion category [13]. The traffic congestion categories are heavy traffic (HT) (vehicle speed 0–20 km/h), moderate (MT) (vehicle speed 20 to 40 km/h), low (LT) (vehicle speed above 40 km/h), and on-sea road (OS), which is the main street located along the sea (vehicle speed above 40 km/h). One area was randomly selected from each category if it contained at least one public hospital. From each selected area, one public hospital was randomly chosen. The on-sea hospital was selected to assess the effect of marine environmental characteristics on the indoor hospital NO₂ levels from traffic sources. 

#### Sample size

The total sample size was four public hospitals in Alexandria, each representing one of the four selected roads. The sample size was determined in accordance with previous studies, which typically included one or two hospitals [14–16]. (Therefore, one hospital was selected to represent each traffic category). The number of indoor locations in each hospital varies depending on hospital activities and the number of departments and rooms.

#### Type of sampling and method of selection

All hospitals included in this study were directly located on one or more main roads, each corresponding to a specific traffic congestion category. The heavy traffic area hospital location (HTH) is located at latitude 31.22º and longitude 29.96º. It is located on a major heavy traffic road and a side street, with a public car park directly in front of its gates. The moderate traffic area hospital (MTH) is located at latitude 31.20º and longitude 29.92º and is directly surrounded by four main roads with moderate traffic. The low traffic area hospital (LTH) is located at latitude 31.27º and longitude 30.03º and bordered by three main low traffic roads. The northern side is adjacent to another healthcare facility. The on-sea road hospital (OSH) is located at latitude 31.21º and longitude 29.88º, in one of Alexandria’s active tourist areas; it is situated directly on the main sea-road. However, despite the area’s overall activity, this section of the road experiences low traffic congestion, while the other sides of the hospital face side roads. The four hospitals were selected in locations with no nearby industrial activities or other NO₂ sources besides traffic. During this study, all hospitals were downwind of the nearby main roads due to their positioning among multiple streets.

### Data collection methods

In this study, the risk of indoor exposure to traffic NO₂ in hospitals during hot and cold months was assessed. Indoor air samples were collected from various hospital locations with different activities during normal daily work hours, excluding areas near kitchens or cafeterias to minimize the indoor NO₂ sources. The sampling systems were placed at the centers of hospital rooms away from any barriers at a height of approximately 1.5 m. Sampling was conducted over 6-hour periods (from 8:00 am to 2:00 pm), representing the morning shifts, the busiest hospital period with the highest work density and peak traffic rush hours (worst-case conditions). Samples were collected from different working areas, including clinics, administrative offices, pharmacies, patient rooms, emergency rooms, intensive care units, operation rooms, and laboratories during normal daily work conditions in both hot and cold months. The air sampling system consisted of a personal pump (Escort ELF personal sampling pump, Zefon International, USA) and standard bubblers (25 ml). Indoor sampling and analysis were conducted following the National Institute for Occupational Safety and Health standard methods (NIOSH 6014) at a flow rate of 0.2 to 1.0 L/min [17].

The NO₂ risk within the hospital’s indoor environment was assessed for the four hospitals using a 3 × 3 risk matrix. The risk level was calculated by multiplying the likelihood and severity scores. The likelihood (probability) of NO₂ exposure was determined based on the calculated or estimated time spent within a hospital room relative to the total shift duration. The Likelihood (L) was classified into 3 classes, with scores ranging from 1 to 3, as indicated in Table 1 [18].

**Table 1:** Likelihood classification of NO₂ exposure in public hospitals

Likelihood score (L)	Percent of presence time of the whole shift	Likelihood Description
1	< 25%	Unlikely
2	25%−75%	likely
3	> 75%	Very likely

**Fig. 1:**
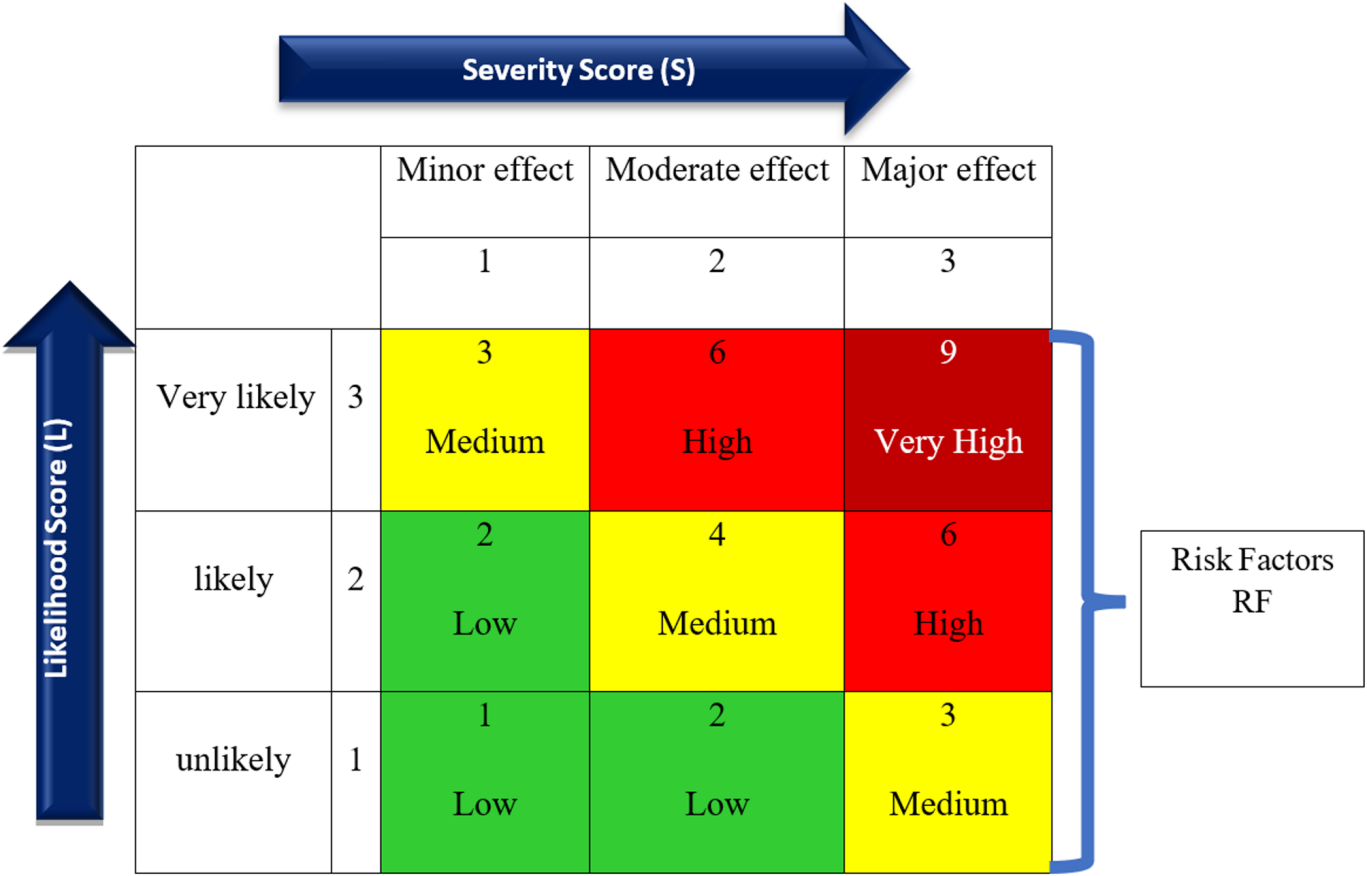
A 3 × 3 risk matrix template used for the calculation of risk level

Severity (S) was also classified into three categories: minor, moderate, and major health effects, as indicated in Table 2. The risk factors (RF) were calculated by multiplying the severity and likelihood scores or by using the risk matrix and color-coding system shown in Fig. 1 [18, 19].

**Table 2:** Severity (S) classification of NO₂ exposure and associated health effects in public hospitals

Severity score (S)	NO₂ Exposure -level (µg/m³) [8]	Health effect	Severity description
1	< 500	• No significant health effects could be detected for healthy people; however, cough, sneeze, and other symptoms may be enhanced for asthmatics [8, 9].	Minor effect
2	500–2000	• Increased prevalence of acute respiratory symptoms. • Decrease in pulmonary functions, especially with exercise (decrease in forced expiratory volume at the 1 st second (FEV1)) • Increased prevalence of respiratory complaints and chest tightness, cough, and chest wheezes, especially in asthmatics [8, 9].	Moderate effect
3	> 2000	• Increased prevalence of acute bronchitis. • Increased prevalence of bronchopneumonia. • Decreased arterial oxygen pressure. • Increased airway resistance manifested by decreased forced vital capacity (FVC), decreased FEV1, and decreased FEV1/FVC ratio (detected by pulmonary function test (PFT)) [8, 9, 20].	Major effect

Accordingly, the risk factors of NO₂ in previous studies were calculated by assuming that the exposure time was either less than or greater than 75%, substituting the likelihood score with 2 or 3. Additionally, the severity score and RF from previous studies were identified based on Table 2 and Fig. 1.

### Statistical analysis

The data were entered into and analyzed using the Statistical Package for Social Sciences (IBM SPSS Statistics), version 21 for Windows. A *p*-value ≤ 0.05 was used as a cut-off point value of statistical significance. The risk factor and NO₂ concentrations were tested for normality using the Kolmogorov-Smirnov Z test. Accordingly, the data were expressed as mean ± standard deviation. A One-Way ANOVA test was used to assess the significance of variation in NO₂ levels across hospitals with different traffic congestion levels. Further analysis using independent-samples t-test revealed a significant difference between each traffic congestion category.

### Quality control

Quality control activities included adhering to the standard operating procedures (SOPs) for sampling, chemical analysis, and data processing. Additionally, air sampling systems, equipment, and spare parts were periodically maintained and calibrated [17, 21].

## Results

This study was conducted during hot and cold seasons at four public hospitals to represent all seasonal variations and activities. A total of 76 in-hospital NO₂ measurements were obtained, distributed as follows: 22 (28.9%) from LTH, 14 (18.4%) from OSH, 18 (23.7%) from MTH, and 22 (28.9%) from HTH. The Kolmogorov-Smirnov test of normality revealed that indoor NO₂ concentrations and risk factors were normally distributed (*p* > 0.05, at C.I.=95%). Therefore, the data were expressed as mean ± standard deviation. The One-Way ANOVA test revealed statistically significant variations (*p* < 0.05, C.I.=95%) in measured indoor NO₂ concentrations across the four hospital types. The Post-HOC Multiple Comparisons LSD test identified a significant difference between NO₂ levels of OSH and both HTH and MTH (*p* < 0.05, C.I.=95%). However, OSH showed nonsignificant variation compared to LTH (*p* > 0.05, C.I.=95%). Moreover, a strong significant direct Spearman’s rho correlation was found between NO₂ levels and traffic congestion (*p* < 0.01, r = + 0.885).

Table 3 presents the indoor NO₂ concentration at the four studied hospitals during hot and cold months. At the Heavy Traffic Hospital (HTH), indoor NO₂ levels during hot months (827.27 ± 39.27 µg/m³) were significantly higher than those recorded during cold months (528.18 ± 32.5 µg/m³) (Independent Samples T-Test for equal variances and means, P-value ≥ 0.05 at 95% C.I). Similarly, for the moderate (MTH), low (LTH), and on-sea (OSH) traffic hospitals indoor NO₂ concentrations were significantly higher during hot [(471.11 ± 6.0 µg/m³), (292.72 ± 22.4 µg/m³), and (367.1 ± 49.9 µg/m³), respectively] compared to cold months [(363.33 ± 5.0 µg/m³), (240.0 ± 18.44 µg/m³), and (242.86 ± 38.61 µg/m³), respectively] (Independent Samples T-Test for equal means).

**Table 3:** Indoor concentration of NO₂ in the four studied hospitals during hot and cold months, Alexandria, Egypt (2020–2021)

Hospital	Months	N^*^	Indoor Concentration of NO₂ (µg/m³)				P-value^∗*^	P-value^∗∗*^
			Mean	S.D.^‡^	Min^§^	Max^♦^		
HTH	Hot	11	827.27	39.27	770.00	930.00	≤ 0.05	≤ 0.05
	Cold	11	528.18	32.50	490.00	600.00		
MTH	Hot	9	471.11	6.01	460.00	480.00	≤ 0.05	
	Cold	9	363.33	5.00	360.00	370.00		
OSH	Hot	7	367.14	49.90	270.00	410.00	≤ 0.05	
	Cold	7	242.86	38.61	170.00	270.00		
LTH	Hot	11	292.73	22.40	270.00	330.00	≤ 0.05	
	Cold	11	240.00	18.44	220.00	270.00		

^*^Number of indoor measurements

^**^t-test for Equality of Means, which is significant at p-value ≤ 0.05 at 95% Confidence Interval

^***^One-Way ANOVA Test, which is significant at p-value ≤ 0.05 at 95% Confidence Interval

^‡^Standard Deviation

^§^Minimum of indoor NO_2_ concentrations

^♦^Maximum of indoor NO_2_ concentrations

The risk factors at HTH ranged from 4 to 6 (yellow: medium risk to red: high risk) during both hot and cold months. In contrast, at MTH, OSH, and LTH, risk factors ranged from 2 to 3 (green: low risk to yellow: medium risk). During hot months at HTH, 72.7% of the studied locations (Table 4) had high-risk factors; these risk factors decreased to 63.6% during the cold months (Table 5). At MTH, OSH, and LTH, moderate risk factors were recorded for 77.8%, 57.1%, and 54.5% of the indoor locations during hot months, with no variation observed in cold months (Tables 4 and 5).

**Table 4:**
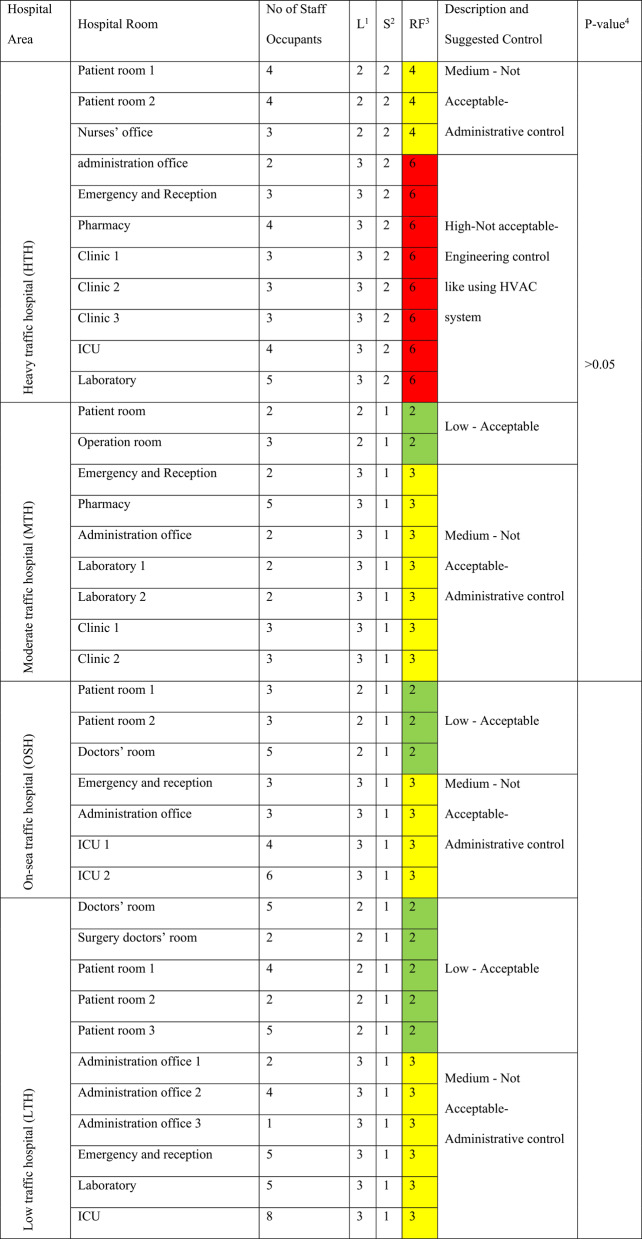
Risk factors of traffic-related nitrogen dioxide at various locations within the four studied hospitals during hot months (2020-2021)

^*^Likelihood Score

^†^Severity Score

^‡^Risk Factor

^§§^One-Way ANOVA test, which is significant at p-value ≤ 0.05 at 95% Confidence Interval

**Table 5:**
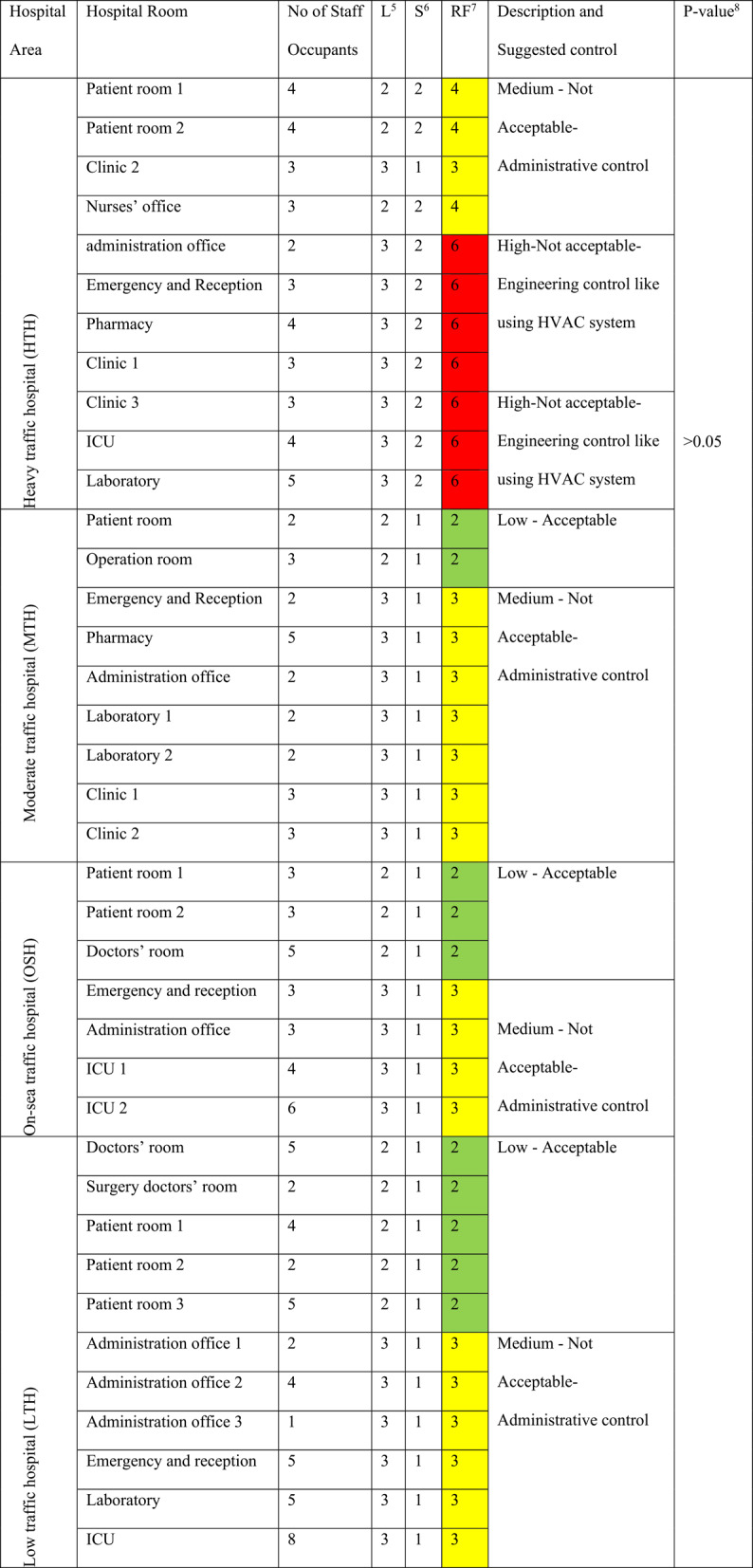
Risk factors of nitrogen dioxide exposures at various locations within the four studied hospitals during cold months (2020-2021)

^*^Likelihood Score

^†^Severity Score

^‡^Risk Factor

^§§^One-Way ANOVA test, which is significant at p-value ≤ 0.05 at 95% Confidence Interval

Tables 4 and 5 presents the risk factors of NO₂ exposure across different indoor locations in the four hospitals. It includes the number of staff occupants at each location during the sampling period, likelihood scores (L) ranging from 1 to 3, severity scores (S) ranging from 1 to 3, and the calculated risk factor (RF) at each location. The risk factor is color-coded as follows: green (low risk, RF = 1–2), yellow (moderate risk, RF = 3–4), bright red (high risk, RF = 6), deep red (very high risk, RF = 9).

A One-Way ANOVA test, followed by Post Hoc Multiple Comparisons LSD Test, revealed a statistically significant variation (*p* < 0.05, C.I.=95%) between the calculated RF levels of the HTH and each of the MTH, LTH, and OSH. However, non-significant variations were observed among MTH, LTH, and OSH (*p* > 0.05, C.I.=95%). The mean RF values at the four hospitals showed no statistically significant differences (*p* > 0.05, C.I.=95%) across different seasons or indoor hospital locations. A Spearman’s rho Correlation Coefficient indicated a strong positive correlation (*p* < 0.01, r = + 0.73) between NO₂ RF and traffic congestion (Table 6).

**Table 6:** The spearman’s Rho correlation between traffic congestion and the calculated risk factor of NO₂

		Risk Factor of NO₂
Traffic congestion	r	+ 0.73
	Sig.	0.00

## Discussion

The present study found that outdoor traffic activities were the primary source of indoor NO₂ levels in the studied hospitals, given that all studied hospitals were downwind of major roads [22]. Indoor concentrations were mainly affected by infiltration and natural ventilation rather than internal sources [23, 24].

The elevated NO₂ levels observed in HTH and the strong positive correlation between NO₂ concentrations and traffic congestion (and lower concentrations in areas of lower traffic congestion) across the four hospitals indicate that indoor NO₂ concentrations in this study were heavily influenced by the outdoor sources, mainly road traffic. This finding aligns with previous research that proved the impact of heavy traffic congestion on increasing levels of indoor NO₂ [25, 26]. Additionally, a study conducted in the Netherlands found that NO₂ concentrations were significantly higher along busy highways compared to less congested roads [26].

This study revealed that NO₂ levels were higher during hot than cold months. This can be attributed to Alexandria’s coastal and tourist nature, which leads to increased traffic congestion during hot months. Additionally, higher temperatures accelerate NO₂ production, potentially contributing to an increase in hospital admissions for respiratory diseases during hot months [27]. This finding aligns with a study in Athens that reported elevated NO₂levels during hot months. The authors explained by citing higher urban activity and increased secondary NO₂ formation due to higher temperatures and increased sunlight [28].Similarly, another study in France found higher NO₂ concentrations during hot months in the Paris region [29]. However,these results contrast with findings of a Turkish study, which found higher indoor and outdoor NO₂ levels in colder months. The increase was primarily due to greater fossil fuel combustion for heating and transportation during winter [30–32]

A Nigerian study examined indoor air pollutant exposures among university laboratory workers and found that mean NO₂ exposure levels exceeded both international and Nigerian air quality guidelines. Furthermore, these NO₂ levels were even higher than those recorded at HTH in the present study. Converting their mean exposure to risk factors at exposure times of less and more than 75% of the shift time, the Nigerian study reported RFs of 6 and 9, indicating high and very high-risk levels. These RF values were comparable to those found in HTH but, in some cases, exceeded them. In contrast, the present study generally found moderate to low RFs in MTH, LTH, and OSH, with only HTH showing most high-risk locations. This suggests that while traffic-related NO₂ exposure in hospitals is concerning, laboratory workers in the Nigerian study faced even greater exposure risks [33].

A 2006 study in Greater Cairo, Egypt, investigated the impact of traffic densities (heavy, moderate, and low) on the indoor air quality of 30 homes. The study found that the average indoor NO₂ levels were higher during hot months compared to cold months across all traffic density categories (heavy, moderate, and low) [34]. These findings align with the pattern observed in the present study, where indoor NO₂ levels increased during hot months. When the RFs were calculated, the Greater Cairo study reported an “unacceptable moderate risk,” which matched the RFs observed in indoor locations at MTH, LTH, and OSH in the present study. However, these RF values were lower than those recorded at HTH, indicating a higher risk level in heavily congested hospital environments compared to residential homes in Cairo.

A study conducted in Saudi Arabia on indoor air quality in a university hospital concluded that outdoor NO₂ levels were the primary source of indoor concentrations [15]. The study reported significantly lower average NO₂ levels compared to all four hospital categories in the present study. When the risk factors (RFs) for the Saudi study were calculated, they ranged from low to moderate, depending on the proposed exposure time. These RF values were comparable to those observed in many indoor locations at MTH, LTH, and OSH in the present study. However, they were not consistent with the higher RF values recorded at HTH, where the impact of heavy traffic congestion resulted in greater NO₂ exposure risks.

### Study Limitation

The aim of this study is limited to the assessment of one traffic-related air pollutant (NO₂ pollutant) and focuses solely on occupational risks, making it unsuitable for evaluating risks to patients, visitors, and escorts.

## Conclusions

This study concluded that indoor NO₂ levels in the studied hospitals were influenced by nearby traffic emissions, as all hospitals were downwind of major roads. Accordingly, NO₂ levels increased with higher traffic congestion. Additionally, NO₂ concentrations were higher during hot months compared to cold months, likely due to increased traffic activity and temperature-related factors. The RFs for most hospital locations in MTH, OSH, and LTH were classified as moderate (requiring administrative controls) or low (acceptable levels of risk). However, in HTH, areas such as the emergency department, clinics, and intensive care unit, which serve hospital staff, patients, and escorts, had high RFs. These areas require engineering controls to reduce exposure, particularly for highly sensitive populations at greater risk of adverse health effects.

These findings are highly significant, as they highlight the impact of traffic-related air pollution on indoor air quality in hospitals, a critical concern for vulnerable patients and healthcare workers. Elevated NO₂ levels, particularly in hospitals located near heavy traffic areas, pose a serious health risk, potentially worsening respiratory conditions and increasing hospital admissions. This study emphasizes the importance of implementing proper ventilation systems and pollution controls in hospitals, especially during periods of high traffic congestion and warmer weather, to minimize exposure and protect public health.

It is recommended to decrease NO₂ exposure among hospital staff and patients; several preventive measures should be implemented. Enhancing mechanical ventilation with suitable and high-efficiency air filtration systems can help limit the infiltration of outdoor pollutants while maintaining indoor air quality. Expanding green spaces around hospitals can further aid in absorbing NO₂ before it enters hospital buildings. Traffic management strategies, such as redirecting traffic away from hospital zones to alternative roads and implementing one-way traffic systems, can help reduce congestion and minimize pollution exposure. Additionally, when constructing new hospitals, selecting upwind locations with low background air pollution, increasing the distance between hospital buildings and major roads, and incorporating green areas can further mitigate exposure to traffic-related pollutants. These measures collectively contribute to protecting hospital occupants from air pollution and improving overall health outcomes.

It is also recommended that future research should expand to include occupational risk assessments of additional traffic pollutants across various hospital locations. Additionally, non-occupational risk assessments should be conducted to better understand the impact of traffic-related air pollution on patients and hospital visitors. Further studies should also incorporate quantitative risk assessment methods to provide a more comprehensive evaluation and allow comparison with the findings of this study.

## Data Availability

All data generated or analyzed during this study will be available upon reasonable request.

## Abbreviations

H-IAQ: Hospital indoor air quality
RA: Risk assessment
NO₂: Nitrogen Dioxide
NIOSH: National Institute of Occupational Safety and Health
PFT: Pulmonary Function Test
FVC: Forced vital capacity
FEV1: Forced expiratory volume in 1 s
HTH: Heavy traffic-congested road hospital
MTH: Moderate traffic-congested road hospital
LTH: Low traffic-congested road hospital
OSH: On -Sea-road hospital
RF: Risk factor
SOPs: Standard operating procedures
